# A new dataset of computed-tomography angiography images for computer-aided detection of pulmonary embolism

**DOI:** 10.1038/sdata.2018.180

**Published:** 2018-09-04

**Authors:** Mojtaba Masoudi, Hamid-Reza Pourreza, Mahdi Saadatmand-Tarzjan, Noushin Eftekhari, Fateme Shafiee Zargar, Masoud Pezeshki Rad

**Affiliations:** 1 Machine Vision Lab, Department of Computer Engineering, Faculty of Engineering, Ferdowsi University of Mashhad, 9177948974 Mashhad, Iran.; 2 Medical Imaging Lab, Department of Electrical Engineering, Faculty of Engineering, Ferdowsi university of Mashhad, 9177948974 Mashhad, Iran; 3 Department of Radiology, Mashhad University of Medical Sciences, Emam Reza Educational, Research, and Treatment Center, 9137913316 Mashhad, Iran

**Keywords:** Biomedical engineering, Embolism

## Abstract

The lack of publicly available datasets of computed-tomography angiography (CTA) images for pulmonary embolism (PE) is a problem felt by physicians and researchers. Although a number of computer-aided detection (CAD) systems have been developed for PE diagnosis, their performance is often evaluated using private datasets. In this paper, we introduce a new public dataset called FUMPE (standing for Ferdowsi University of Mashhad's PE dataset) which consists of three-dimensional PE-CTA images of 35 different subjects with 8792 slices in total. For each benchmark image, two expert radiologists provided the ground-truth with the assistance of a semi-automated image processing software tool. FUMPE is a challenging benchmark for CAD methods because of the large number (i.e., 3438) of PE regions and, more especially, because of the location of most of them (i.e., 67%) in lung peripheral arteries. Moreover, due to the reporting of the Qanadli score for each PE-CTA image, FUMPE is the first public dataset which can be used for the analysis of mortality and morbidity risks associated with PE. We also report some complementary prognosis information for each subject.

## Background & Summary

Pulmonary embolism (PE) is a sudden blockage of a lung artery by a deep vein thrombosis (DVT) clot, typically originating in the pelvis veins and carried by the blood flow through the heart into the lung. Since it may reduce respiratory capability by pulmonary artery (PA) closure, early diagnosis and treatment of DVT can decrease the risk of PE.

However, once arterial obstruction exceeds 50% of the cross-sectional area, massive PE may occur with acute and severe cardiopulmonary failure because of right ventricular overload. It was reported that 70% of patients died within the first hour after onset of the above symptoms. Therefore, early and precise diagnosis of PE is important, due to the high morbidity and mortality risk^1,2^.

Contrast-enhanced computed tomography (called CT angiography or CTA) images have been widely used for PE diagnosis^3–5^ because of their suitable lesion discrimination in blood vessels^6^. Specifically, PE regions appear as dark spots among the bright regions of blood arteries in CTA images^7^. The radiologist should record the CTA image in a suitable time interval after injection of the contrast material and before its traveling from the arteries to the veins. In this case, although the vein and PE regions may have similar gray-levels in the CTA image, the latter can be distinguished from the former by its higher contrast. Nevertheless, lymphatic tissue, parenchymal disease, and partial volume effect may also provide similar dark regions (especially, on artery boundaries) in CTA images^8^. This is why the manual delineation of PE regions is a time consuming task and depends on the expert insight^9^.

In recent years, by progressing computing and computational technologies, computer-aided detection (CAD) systems have gained increasing impact in clinical and research applications^5^. However, due to the above challenges, automated/semi-automated detection of PE, still, is a challenging endeavor for radiologists, physicians, and biomedical engineers. These groups are unable to precisely evaluate and compare their results with each other, due to the lack of a proper dataset of PE-CTA images with suitable ground-truth, evaluation scores, and prognosis information. To tackle this problem, some researchers have generated private datasets, which are not widely shared^4,8,10^. Recently, Madrid-MIT M+Visión Consortium^11^ supplied a public dataset of 20 PE-CTA images with ground-truth. However, they reported neither the clinical information of subjects nor, evaluation scores of PE-CTA images.

In this paper, we present a new dataset of three dimensional (3D) PE-CTA images, called FUMPE (standing for Ferdowsi University of Mashhad's PE dataset), for computer-aided detection with research and education purposes. It includes 35 PE-CTA images with a total of 8792 slices. Furthermore, an expert radiologistmanually and precisely delineated the PE regions in every slice of each CTA image as the ground-truth. We took advantage of a semi-automated software tool to enhance the segmentation results. The final PE regions were re-examined by another expert radiologist. In addition, for further evaluation, the first radiologistprovided five CTA measurements for every benchmark image.

## Methods

**Figure 1:**
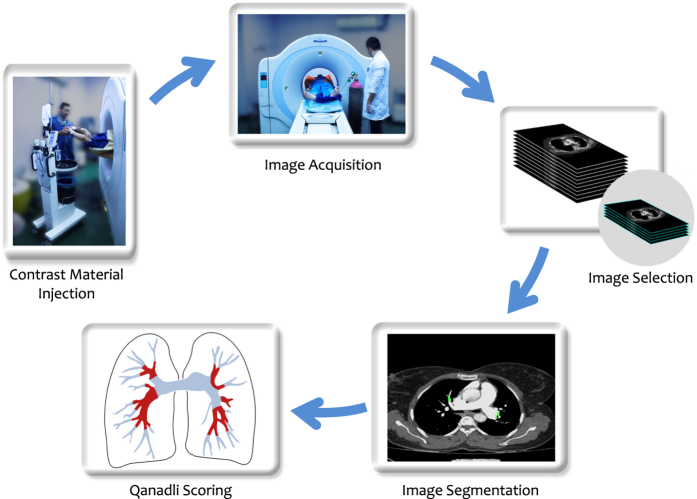
Five steps of the development process of the proposed dataset.

We primarily obtained ethical approval of the ethics committee of Mashhad University of Medical Sciences. Although all images have been anonymously published in the proposed dataset to avoid the risk of privacy breach, we got a signed informed consent from every patient. As shown in Fig. 1, the development process of the proposed dataset consists of contrast material injection, image acquisition, image selection, image segmentation, and Qanadli scoring, as comprehensively stated in the sequel.

### Contrast material injection

In a normal PE-CTA, the pulmonary arteries should be full of the contrast material while the aorta should be empty of it. Therefore, a total of 70-100 mL of non-ionic contrast material (containing 300-370 milligrams of iodine per milliliter) was injected into the right antecubital vein by using gauge-18 or -16 catheters (with the flow of 4-5 mm per second) at 10-12 seconds before imaging.

### Prognosis symptoms

To collect the FUMPE dataset, 400 PE-CTA images were primarily recorded from different patients. The most-common patient-complaints were dyspnea, tachypnea, and pleuritic chest pain with haemoptysis. Moreover, some patients had non-specific signs and symptoms, such as tachycardia, palpitations, wheezing, and cough. However, patients with massive PE had hypotension, extreme hypoxemia, cyanosis, syncope, or even cardiac arrest. Furthermore, for non-urgent patients, the DVT test was performed.

### Imaging

CT-scanning was performed in Emam-Reza and Ghaem Medical Centers (http://quaem.mums.ac.ir/index.php/en) by using the NeuViz 16 multi–slice helical CT scanners of *Philips and Neusoft Medical System Co., Ltd* with 120 kVp, 0.75 mm × 16 collimation, the gantry rotation time of 0.75 s, and a beam-pitch of 1.2. Also, in order to automatically adjust the tube current, scanners took advantages of both the dose modulation and angular/longitudinal tube-current modulation (with automatic current selection) for all subjects except Patient21. The range of the tube current variations for each subject was reported in Table 1. All PE-CTA images of FUMPE were acquired in one breath hold with:

- slice-thickness≤1mm (except for Patient24 and Patient32 with slice-thickness=2mm)
- slice-interval≤1.5 (except for Patient03, Patient10, and Patient28 with slice-interval=4mm)
- in the caudocranial direction (except for Patient12 and Patient13 with the craniocaudal direction)

### Image selection

It was frequently demonstrated that CAD systems could better extract PE regions in the main arteries compared to the peripheral vessels, due to higher contrast and better discrimination^8^. Thus, a suitable benchmark dataset for evaluation of CAD systems should considerably include a large number of PE regions in the peripheral arteries. Therefore, from among all the recorded PE-CTA images, by visual inspection, we choose 35 images with the largest number of PE regions in peripheral arteries to make the proposed dataset.

### Image segmentation

To establish the ground-truth, a board-certified radiologist (with over 5-year experience for PE-CTA analysis) primarily delineated all PE regions of interest (PE-ROIs) in each PE-CTA image. He also took advantage of a semi-automated software tool called MIS (standing for medical image segmenter) which supports the coronal and sagittal reconstructions (in addition to the original axial view) to ensure about delineation accuracy. Finally, the delineated PE-ROIs were re-examined and approved by the head of the radiology department (with 18-year experience) of Emam-Reza Medical Center (http://emamreza.mums.ac.ir/index.php/en).

### Code availability

We developed the MIS software tool in the MATLAB R2017 environment. It consists of a GUI window in which the user can see a 3D DICOM image in the axial, coronal, and sagittal views. Also, the user can choose the region of interest in each slice by multiple mouse selections. The software took advantage of a semi-automated segmentation algorithm which consists of the thresholding and connected-component analysis steps^12^. It can determine the local connected region to a seed point, chosen by the user, through a gray-level similarity criterion. The source codes (in the MATLAB environment), compiled executable file, and pictorial user manual of MIS are publicly available in: https://doi.org/10.6084/m9.figshare.6289085 (with the Figshare Repository).

### CTA measurements

**Figure 2:**
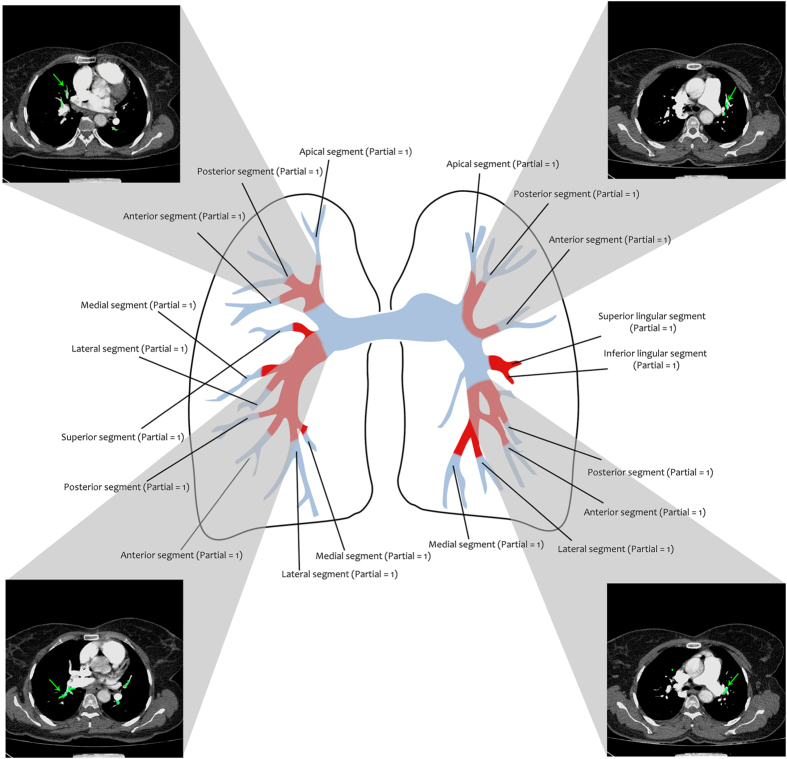
Sample Q-Score computation. The arterial segments of the left and right lungs of Patient16 (used for Q-score computation) are illustrated as an example. The obstruction index of each segment is indicated in the figure. The total Q-score (i.e. 19) was computed as the superposition of all the obstruction indices.

We provided five measurements for each PE-CTA image, as follows:

- *RV/LV Ratio*: The right ventricular (RV) failure is one of the most important causes of early death after PE^13^. CTA enables the radiologist to assess RV dysfunction by calculating the ratio of RV to left ventricular (LV) diameter (called RV/LV ratio) in the reconstructed four-chamber views.
- *Reflux into IVC*: Reflux of the contrast material into the inferior vena cava (IVC), which can be observed in CTA images, is associated with right heart failure due to PE^14^.
- *Straight Septum* & *PA Diameter*: Severe PE increases the right heart pressure. In this case, the interventricular septum may be abnormally shifted toward the left ventricle^15^; and also, the diameter of the main pulmonary artery (lateral to the ascending aorta and at the level of its bifurcation) may be increased^16^.
- *Q-score*: After image segmentation, we assessed the arterial clots of each subject according to the Qanadli scoring system (Q-score). Generally, the Q-score is computed as the superposition $Q=\sum_{k=1}^{n}d_{k}$ where *n* indicates the total number of proximal clot sites and *d*_*k*_ determines the obstruction index of the *k*-th one. In more detail, in the left lung, the upper, lingual, and lower lobar arteries are branched into three (apical, posterior, and anterior), two (superior and inferior), and five (superior, medial, lateral, posterior, and anterior) segments, respectively. Similarly, the lobar arteries of the right lung are also separated into 10 segments, in the same manner. Thus, as illustrated in Fig. 2, we totally have *n*=20 segments in both lungs. For the *k*-th segment (*k*=1,2,…,*n*), *d*_*k*_ is set equal to 0, 1, and 2 for the clot-free, partial obstruction, and total occlusion situations, respectively^17^. Once there is an embolus in the most proximal arterial level, its corresponding index is computed as the superposition of the obstruction indices of all segmental arteries arising distally. For example, Fig. 2 illustrates the obstruction indices of all arterial segments in the left and right lungs of Patient16. The Q-score can be used for prognosis evaluation, treatment-reply, and determining the anti-coagulant treatment period^18^. Also, the patients with larger Q-scores than 18 have high mortality and morbidity rates^19^.

## Data Records

All data records described in this paper are available on the Figshare Repository, organized in 35 different patients (Patient01 to Patient35, Data Citation 1) and one ground-truth archive (Ground Truth, Data Citation 1). Each patient archive includes all slices of the corresponding 3D CTA image (stored in the DICOM file format) while the ground-truth archive consists of all the 3D ground-truth images of FUMPE in the MAT file format. MAT files can be simply loaded to the MATLAB programming environment by using the function *load*. In every ground-truth image, the foreground and background voxels were indicated by the gray-levels 1 and 0, respectively.

## Technical Validation

Each image was visually checked by an experienced CT technologist to be artifact-free and have sufficient contrast for image analysis. If the image quality was not acceptable, he repeated the image acquisition process.

### Summary of the dataset

Table 1 reports the characteristics of all CTA images of the proposed dataset including the subject gender and age, DVT test, slice thickness and interval, range of the tube current, imaging direction, number of slices, number of PE regions in the main arteries, and that in the peripheral arteries. FUMPE includes the PE-CTA images of 17 male and 18 female patients (aged 24-82 years). In addition, from among all FUMPE images (with totally 8792 slices), only Patient24 and Patient32 have no PE-clots (false positives) in both the main and peripheral arteries.

**Figure 3:**
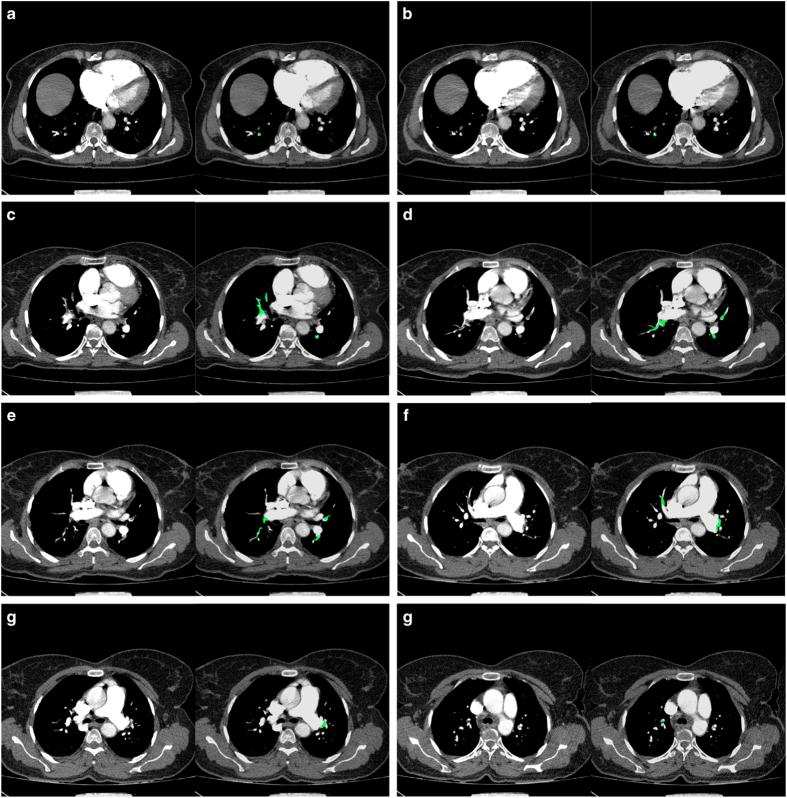
A sample CTA image of FUMPE. Including the source (left-hand side in each pair) and ground-truth (right-hand side in the same pair) images corresponding to the (**a**) 77th, (**b**) 80th, (**c**) 106th, (**d**) 116th, (**e**) 119th, (**f**) 133th, (**g**) 139th, and (**h**) 151th slices of Patient16.

For example, Fig. 3 illustrates the source and ground-truth images of the 77th, 80th, 106th, 116th, 119th, 133th, 139th, and 151th slices of Patient16. Note that here, all PE regions of the ground-truth were indicated by the semi-transparent green color over the source image for better visual inspection. Also, the size of PE regions was significantly various from few to hundreds of voxels.

For every ground-truth image of the dataset, we counted the number of PE regions in the main and peripheral arteries. As reported in Table 1, the proposed dataset totally includes 3438 PE-ROIs; such that most of them (i.e. 67%) are located in the peripheral arteries. Therefore, FUMPE is a challenging benchmark for evaluation of CAD systems.

**Figure 4:**
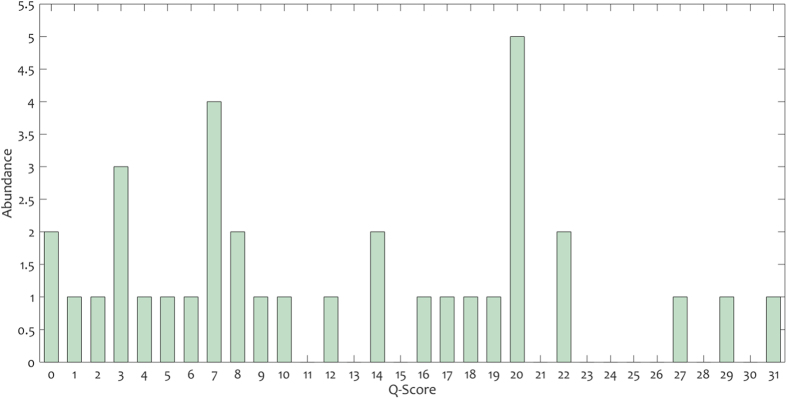
The Q-score histogram of FUMPE.

Finally, Table 2 reports the five specified CTA measurements (including RV/LV ratio, Reflux into IVC, straight septum, PA diameter, and Q-score) for all FUMPE images. As further illustrated in Fig. 4-scores were ranged from 0 to 31. Also, the most frequent Q-scores were 20, 7, and 3, with the abundance of 5, 4, and 3 subjects, respectively. Moreover, 11 patients, with larger Q-scores than 18, had high mortality/morbidity risk.

### Comparing with other PE datasets

As shown in Table 3, FUMPE is further compared with 14 PE-CTA datasets reported in^3–5,8,10,11,20–27^. All the counterpart datasets, except m+visión^11^, are private (i.e. with non-public accessibility). Clearly, FUMPE includes a large number of PE-ROIs compared to the other datasets. Furthermore, it is the first public PE dataset with the Q-score evaluation, which can be used for development of automatic scoring algorithms and medical education purposes. It is also the only dataset which provides appropriate complementary prognosis information such as DVT test, RV/LV ratio, reflux into IVC, straight septum, and PA diameter.

## Additional information

**How to cite this article**: Masoudi, M. *et al*. A new dataset of computed-tomography angiography images for computer-aided detection of pulmonary embolism. *Sci. Data* 5:180180 doi: 10.1038/sdata.2018.178 (2018).

**Publisher’s note**: Springer Nature remains neutral with regard to jurisdictional claims in published maps and institutional affiliations.

## Supplementary Material



## Figures and Tables

**Table 1:** Different characteristics of CTA images reported in FUMPE.

**Case**	**Gender**	**Age**	**DVT test**	**Slice Thickness (Interval)**	**Tube Current (mA)**	**Imaging Direction**	**#Slice**	**#PE-ROIs (Main)**	**#PE-ROIs (Peripheral)**	**#PE-ROIs (Total)**
Patient01	M	32	X	1.0 (1.0)	[198, 297]	Caudocranial	201	46	34	80
Patient02	F	70	✓	1.0 (1.0)	[123, 155]	Caudocranial	185	0	21	21
Patient03	M	42	NA	1.0 (4.0)	[210, 282]	Caudocranial	210	39	55	94
Patient04	F	70	X	1.0 (1.0)	[183, 239]	Caudocranial	197	17	29	46
Patient05	F	NR	NA	1.0 (1.0)	[147, 210]	Caudocranial	217	71	92	163
Patient06	F	NR	NA	1.0 (1.0)	[135, 167]	Caudocranial	232	0	15	15
Patient07	M	52	✓	1.0 (1.0)	[196, 251]	Caudocranial	197	95	147	242
Patient08	M	28	✓	1.0 (1.0)	[160, 257]	Caudocranial	273	23	36	59
Patient09	F	69	NA	1.0 (1.0)	[124, 184]	Caudocranial	237	0	53	53
Patient10	M	71	NA	1.0 (4.0)	[142, 224]	Caudocranial	204	0	8	8
Patient11	F	63	NA	1.0 (1.0)	[60, 87]	Caudocranial	217	0	18	18
Patient12	F	71	✓	1.0 (1.0)	[130, 160]	*Craniocaudal*	178	0	77	77
Patient13	M	27	✓	1.0 (1.0)	[235, 303]	Caudocranial	189	30	23	53
Patient14	F	29	NA	1.0 (1.0)	[231, 281]	Caudocranial	217	0	98	98
Patient15	M	61	✓	1.0 (1.0)	[145, 204]	Caudocranial	250	15	31	46
Patient16	F	53	NA	1.0 (1.0)	[142, 236]	Caudocranial	235	18	59	77
Patient17	F	62	✓	1.0 (0.5)	[232, 325]	Caudocranial	475	194	60	254
Patient18	M	82	✓	1.0 (0.5)	[165, 295]	Caudocranial	452	54	102	156
Patient19	M	76	NA	1.0 (1.0)	[206, 266]	Caudocranial	370	51	300	351
Patient20	M	39	NA	1.0 (0.5)	[168, 321]	Caudocranial	424	19	48	67
Patient21	M	80	NA	1.0 (1.0)	242	Caudocranial	217	51	0	51
Patient22	F	80	NA	1.0 (1.0)	[174, 246]	Caudocranial	185	0	22	22
Patient23	M	54	✓	1.0 (1.0)	[199, 276]	Caudocranial	297	0	116	116
Patient24	F	31	NA	2.0 (1.5)	[121, 175]	Caudocranial	139	0	0	0
Patient25	F	33	NA	1.0 (1.0)	[184, 285]	Caudocranial	221	0	67	67
Patient26	F	24	NA	1.0 (1.0)	[134, 283]	Caudocranial	213	77	131	208
Patient27	M	28	✓	1.0 (1.0)	[107, 207]	Caudocranial	277	53	88	141
Patient28	F	31	✓	1.0 (4.0)	[182, 264]	Caudocranial	183	19	0	19
Patient29	M	70	NA	1.0 (1.0)	[152, 203]	Caudocranial	197	24	72	96
Patient30	F	77	✓	1.0 (1.0)	[120, 228]	Caudocranial	205	35	64	99
Patient31	M	70	✓	1.0 (1.0)	[144, 227]	*Craniocaudal*	268	54	103	157
Patient32	F	74	NA	2.0 (1.5)	[106, 132]	Caudocranial	155	0	0	0
Patient33	F	72	✓	1.0 (0.5)	[192, 277]	Caudocranial	435	115	60	175
Patient34	M	80	NA	1.0 (1.0)	[182, 279]	Caudocranial	189	34	121	155
Patient35	M	66	NA	1.0 (0.5)	[217, 328]	Caudocranial	451	0	154	154
						Total	8792	1134 (33%)	2304 (67%)	3438
Including the subject gender and age, deep vein thrombosis (DVT) test, slice thickness and interval, tube current, imaging direction, total number of slices (#Slice), total number of regions of interest with pulmonary embolism (#PE-ROIs) in main arteries, #PE-ROIs in peripheral arteries, and #PE-ROIs in the total arteries.										

**Table 2:** Five different measurements reported for each CTA image of FUMPE.

**Case**	**RV/LV ratio**	**Reflux into IVC**	**Straight Septum**	**PA Diameter**	**Q-Score**
Patient01	0.86 (=31/35)	X	X	33	20
Patient02	1.13 (=27/24)	✓	X	24	1
Patient03	1.03 (=33/32)	X	✓	33	20
Patient04	1.33 (=24/18)	✓	✓	31	29
Patient05	0.89 (=42/47)	X	X	28	20
Patient06	1.03 (=31/30)	✓	X	38	3
Patient07	2.00 (=40/20)	✓	✓	31	31
Patient08	1.04 (=25/24)	X	X	30	7
Patient09	1.18 (=20/17)	✓	X	30	4
Patient10	0.78 (=21/27)	X	X	26	7
Patient11	0.72 (=23/32)	X	X	27	2
Patient12	0.75 (=24/32)	X	X	28	6
Patient13	0.65 (=20/31)	✓	✓	27	14
Patient14	0.73 (=22/30)	✓	X	25	8
Patient15	0.87 (=27/31)	X	X	24	8
Patient16	1.65 (=28/17)	✓	✓	28	19
Patient17	1.46 (=41/28)	✓	✓	37	20
Patient18	1.19 (=25/21)	✓	X	22	12
Patient19	1.47 (=22/15)	✓	X	24	16
Patient20	0.75 (=12/16)	X	X	19	5
Patient21	1.56 (=67/43)	✓	✓	36	10
Patient22	0.93 (=27/29)	X	X	28	3
Patient23	1.12 (=28/25)	✓	✓	41	17
Patient24	0.97 (=38/39)	✓	X	25	0
Patient25	0.40 (=19/48)	X	X	31	3
Patient26	1.65 (=28/17)	✓	✓	32	27
Patient27	0.81 (=17/21)	X	X	20	18
Patient28	0.59 (=23/39)	✓	X	25	7
Patient29	1.30 (=39/30)	✓	✓	27	22
Patient30	0.63 (=25/40)	X	X	32	7
Patient31	1.19 (=32/27)	✓	X	23	20
Patient32	1.21 (=34/28)	✓	X	28	0
Patient33	1.33 (=20/15)	✓	✓	26	14
Patient34	1.94 (=35/18)	✓	✓	27	22
Patient35	0.91 (=29/32)	X	X	26	9
Including the ratio of right ventricular to left ventricular diameter (RV/LV ratio), reflux into the inferior vena cava (IVC), straight septum, pulmonary artery (PA) diameter, and Qanadli score (Q-Score).					

**Table 3:** Comparing FUMPE with 14 different PE-CTA datasets.

**PE-CTA Dataset**	**#Subjects**	**#Clots**	**#PE-ROIs**	**Public Accessibility**	**Available Scoring**	**Prognosis Information**
Masutani et al.^20^	11	21	−	X	X	X
Pichon et al.^21^	3	22	−	X	X	X
Das et al.^22^	33	168	−	X	X	X
Digumarthy et al.^23^	39	270	−	X	X	X
Maizlin et al.^24^	8	45	−	X	X	X
Kiraly et al.^25^	8	69	−	X	X	X
Zhou et al.^26^	14	225	−	X	X	X
Buhmann et al.^3^	40	352	−	X	X	X
Wittenberg et al.^4^	119	38	−	X	X	X
Bouma et al.^8^	19	116	−	X	X	X
Özkan et al.^5^	33	450	−	X	X	X
Park et al.^10^	20	44	648	X	X	X
Tajbakhsh et al.^27^	121	326	−	X	X	X
m+visión^11^	20	105	5521	✓	X	X
FUMPE	35	119	3438	✓	Q-score	DVT Test, RV/LV Ratio, Reflux into IVC, Straight Septum, PA Diameter
In terms of the number of subjects (#Subjects), number of clots (#Clots), number of regions of interest with pulmonary embolism (#PE-ROIs), public accessibility, available scoring, and prognosis information.						
